# First indoor radon mapping and assessment excess lifetime cancer risk in Iran

**DOI:** 10.1016/j.mex.2019.09.028

**Published:** 2019-09-30

**Authors:** Samira Sherafat, Sepideh Nemati Mansour, Mohammad Mosaferi, Nayyereh Aminisani, Zabihollah Yousefi, Shahram Maleki

**Affiliations:** a Health Faculty, Student Research Committee, Tabriz University of Medical Sciences, Tabriz, Iran; b Health and Environment Research Center, Tabriz University of Medical Sciences, Tabriz, Iran; c Tabriz Health Services Management Research Center, Tabriz University of Medical Sciences, Tabriz, Iran; d Noncommunicable Diseases Research Center, Neyshabur University of Medical Sciences, Neyshabur, Iran; e Department of Environmental Health Engineering, Faculty of Health and Health Sciences Research Center, Mazandaran University of Medical Sciences, Sari, Iran; f Medical Geography, Ministry of Health, Tehran, Iran

**Keywords:** Indoor radon mapping and health risk assessment, Radon map, Annual effective dose, Excess lifetime cancer risk, Floor, CR-39

## Abstract

Radon (222Rn) is believed to be the main contributor to lung cancer second to smoking. The first national indoor radon map derived from some scattered regional radon surveys in Iran.

The arithmetic mean of indoor radon concentration was calculated to 117.4 ± 97.7 Bq/m^3^. The mean excess life time cancer risk (ELCR) values were found to be in the range of 0.1%–4.26%, with an overall average value of 1.01%. The mean radon-induced lung cancer risk was 46.8 per million persons. Absence of sufficient indoor radon data showed that national wide monitoring programs should be activated in uncovered areas.

Meanwhile, in order to provide further baseline values for radon mapping, we attempted to survey the radon levels inside 50 dwellings of Shabestar County in northwest of Iran. The investigation was also focused on the effects of some buildings related variables. The radon levels recorded varied from 3.92 to 520.12 Bq/m^3^, with a mean value of 56.19 ± 45.96 Bq/m^3^. In 9% of dwellings radon concentration exceeded 100 Bq/m^3^, the limit recommended by the World Health Organization. The average annual effective dose received by the residents of studied area was calculated to be 1.4 mSv. The ELCR was estimated to be 0.54%.

## Graphical abstract

**Figure:**
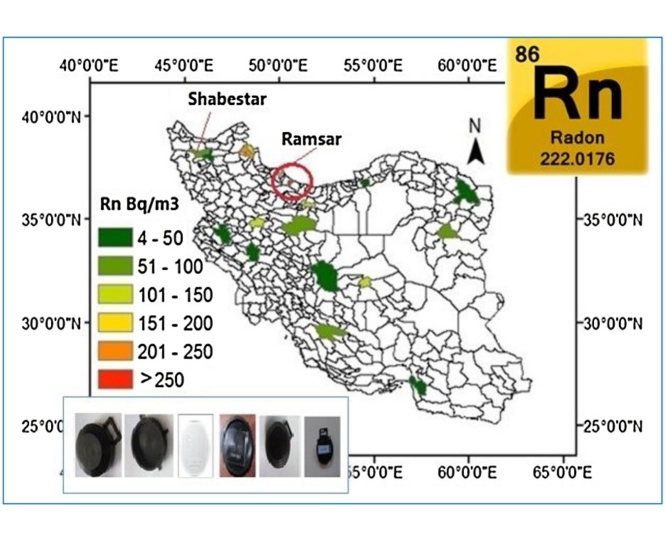

**Specification Table**

**Table:** 

Subject Area:	Environmental Science
More specific subject area:	Indoor radon
Protocol name:	Indoor radon mapping and health risk assessment
Reagents/tools:	solid-state nuclear detectors of SSNTDs type CR-39, Radon mapping by ARC GIS (Ver. 10.3)
How data were acquired:	All available data relevant to indoor radon surveys across country up to 2019 were collected and used in mapping. CR-39 detectors after three-month exposure in dwellings of Shabestar county were analyzed to determine radon concentration according to the U.S. EPA protocol [1].
Trial registration:	Not applicable
Ethics:	Not applicable

**Value of the Protocol**

**Table:** 

• We reviewed and summarized all researches conducted on the levels of indoor radon and provided first radon map in Iran based on the published papers. • Public exposure database in terms of effective dose, ELCR and risk of lung cancer was prepared.

## Description of protocol

Radon is a colorless, odorless and tasteless natural radioactive gas. It is a product of the degradation of uranium and has a radioactive half-life of about four days. Prolonged exposure to elevated radon concentrations has been linked to an increased lung cancer risk [2].

High background radon radiation levels has been reported in Guarapari, Brazil; Kerala, India; Yangjiang, China ; Ramsar, Iran [[3], [4], [5]]. However, not all areas have a high radon concentration and there is no way to know the radon level and consequently possible risk in a specific site before testing. Radon concentration varies across the country, so easy, cost-effective and reliable measuring radon levels at dwellings of different areas can be used to develop a national database including maps of residential radon exposure.

In Iran, Radon gas measurement was considered by the Iranian Ministry of Health since 2013 in the framework of the National Radon Measuring Plan. Subsequently, in different provinces and cities of Iran, the program has been implemented sporadically and the results of these monitoring are published in the form of scientific papers. For example, the most recent researches carried out in Northern Iran [4], Central Iran [5], Tehran [6], Tabriz [7], Isfahan [8], Shiraz [9], Mashhad [10], Hamedan [11], Ramsar [12], Yazd [13], Qom [14], Kermanshah [15], Khoram Abad [16], and Minab [17] can be mentioned. Despite some scattered regional indoor radon surveys in Iran, radon mapping has not been carried out yet to increase awareness of the hazards of exposure to radon and to target future radon surveys. So, the main objectives of this study was review and summarize all researches conducted on the levels of indoor radon and also provide map of radon concentration across the country based on the published papers.

Besides, we have tried to measurement of indoor radon levels in Shabestar residential homes in the East Azerbaijan-northwest of Iran as a case survey along with analyzing the factors influencing the concentrations. A little information on indoor radon activity in Azerbaijan district is available in literature. The results of this study could be useful in developing the radon map of Iran.

## Development a first trial radon map

All available data relevant to indoor radon surveys across country up to 2019 was collected and used in mapping and producing database. The map includes data from 20 cities displaying the levels of indoor radon activity in 3441 dwellings in Iran.

**Fig. 1:**
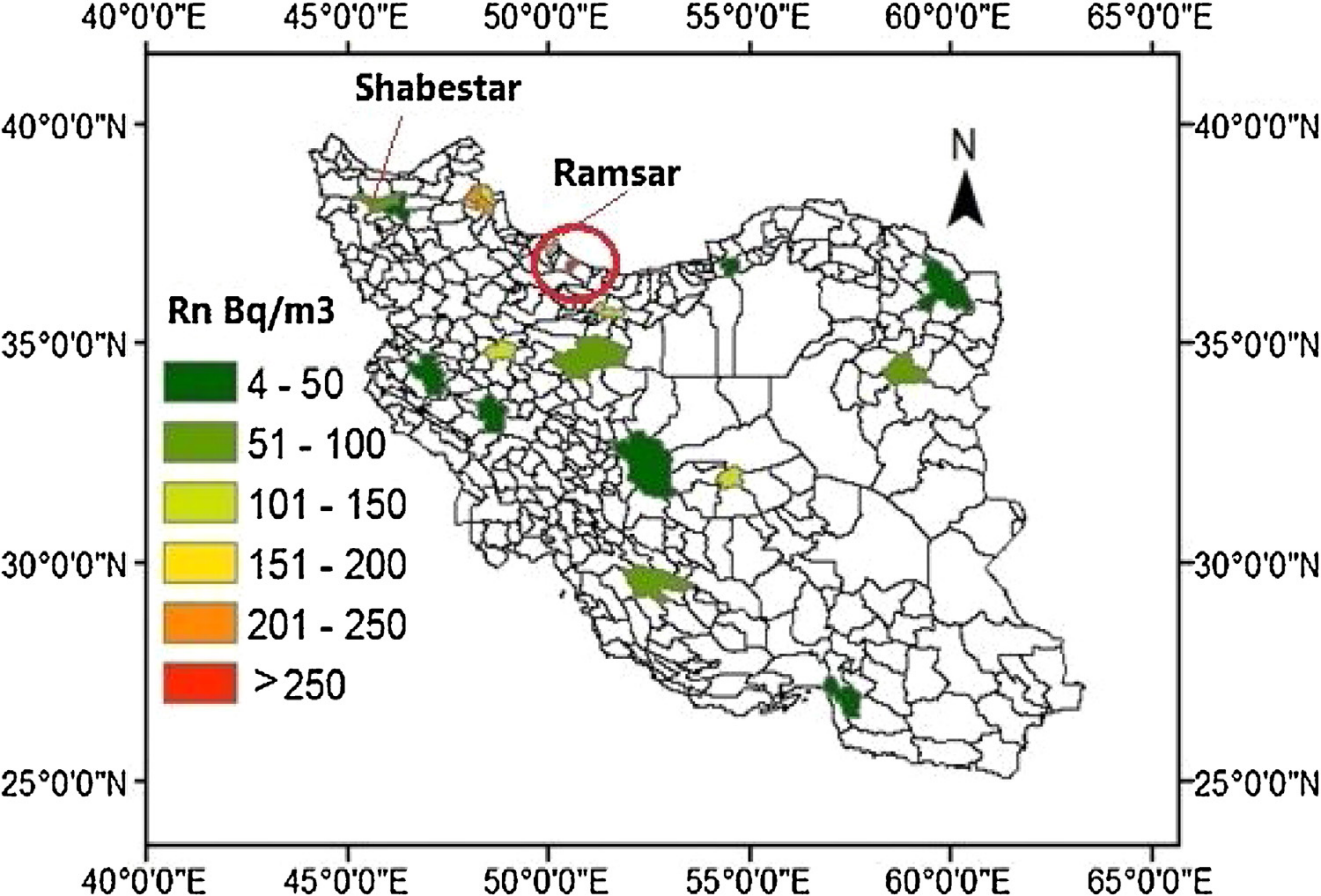
Indoor radon activity map of Iran.

Fig. 1 depicts the map of mean indoor radon concentration values in Iran. Our database including indoor radon levels and associated radiological parameters are given in Table 1 as well. However, only data from 20 cities representing 4.5% of the Iranian cities with population more than 20,000 people was available.

**Table 1:** Indoor radon concentration studies in different cities of Iran.

Region	Number of dwellings	222Rn (Bq/m^3^)		Mean Effective dose (mSv/y)	ELCR	LCC×10^−6^	Excessive rate (%)	Ref
		Mean(SD)	(Min, Max)					
Ramsar(1)	500	Autumn:355 Winter:476	(Max:31,080)	Autumn: 8.95 Winter: 12	3.44 × 10^−2^ 4.6 × 10^−2^	161.11 216	–	[35]
Ramsar(2)	85	578(677)	–	17.6	6.7 × 10^−2^	316.18	45% between 400-3200 Bq/m^3^ 15%>100 Bq/m^3^	[36]
Babolsar	14	88(35)	–	2.68	1.03 × 10^−2^	48.42		
Gonabad	27	84(31)	–	2.56	9.86 × 10^−3^	46.08		
Tehran(1)	80	80(84)	–	2.44	9.3 × 10^−3^	43.94		
Tehran(2)	30	104	(31,460.2)	2.62	1 × 10-2	47.16	38%>100 Bq/m^3^	[6]
Lahijan	400	163(57)	–	3.43	1.3 × 10-2	61.74	In a majority of dwellings >100 Bq/m^3^ Ave:163 Bq/m^3^ Max:2386 Bq/m^3^ (Ardabil), Min:55 Bq/m^3^(Lahijan)	[4]
Ardabil	400	238(24)	–	5	1.9 × 10-2	90		
Namin	176	144(73)	–	3.63	1.4 × 10-2	65.34		
Sar-Ein	148	159(116)	–	4	1.54 × 10^−2^	72		
Khorramabad	56	43.4(40.37)	(1.08,196.8)	1.09	4.2 × 10^−3^	19.62	10.1%>100 Bq/m^3^	[16]
Qom	123	95.83	(15,259)	2.41	9.2 × 10^−3^	43.38	24.3%>100 Bq/m^3^	[14]
Shiraz(1)	262	94(52)	(17.4, 280.7)	2.37	9.1 × 10^−3^	42.66	–	[37]
Shiraz(2)	185	57.6 (33.06)	(17,250)	1.45	5.6 × 10^−3^	26.1	5.4%>100 Bq/m^3^	[9]
Kermanshah	102 (hospitals)	11.4(4.9)	–	0.28	1 × 10^−3^	5.04	–	[15]
Minab	34	33.7	(8,67)	0.85	3.2 × 10^−3^	15.3	–	[17]
Isfahan	51	28.57(39.38)	(3,251)	0.72	2.7 × 10^−3^	12.96	4%>100 Bq/m^3^	[8]
Mashhad	150	31.9	(12.3, 135.2)	0.8	3 × 10^−3^	14.4	5.3%>100 Bq/m^3^	[10]
Hamadan	70	108	(4,364)	2.72	1 × 10^−2^	48.96	–	[11]
Yazd	84	137.4(149.5)	(5.55,747.4)	3.46	1.3 × 10-2	62.28	30% of basements >148 Bq/m3	[13]
Gorgan	218	43.99(37.8)	–	1.1	4.2 × 10-3	19.8	3%>148 Bq/m^3^	[38]
Tabriz	196	39(25)	–	0.98	3.7 × 10-3	17.64	–	[7]
Shabestar County	50	56.19(45.96)	(3.92,520.1)	1.4	5.4 × 10-3	25.2	10%>100 Bq/m^3^	(Present study)
Total	3441	G.M:72.05 A.M:117.4	–	Mean: 2.6	Mean: 1 × 10^−2^	Mean: 46.8	–	

ELCR = Excess Life Time Cancer risk, G.M = Geometrical mean, A.M = Arithmetical mean.

As shown in Table 1 and in indoor radon activity map (Fig. 1), the mean indoor radon concentration levels in most cities are below the WHO action level and no more than 10% of them have radon concentration exceeding 200 Bq/m^3^. The geometric mean of radon concentration was calculated to 72.05 Bq/m^3^. But anyway, many areas of country are still not covered by this map and further surveys should be carefully designed.

Table 2 displays the comparison of global indoor radon concentrations in different countries with the results of the present study. The residential radon value in Iran is lower than Romania and Jordan.

**Table 2:** Comparison of indoor radon levels in Iran with some others countries.

Countries	Concentration of indoor radon (Bq/m^3^)	Ref
Iran	117.4	Present study
Azerbaijan	84	[39]
Turkey	81	[28]
Iraq (Baghdad)	116	[40]
Pakistan (Azad Kashmir district)	100	[41]
Lebanon	23.5	[42]
Oman	21	[43]
Saudi Arabia (West &Southwest regions)	32	[44]
Japan	14.3	[45]
South Korea	53	[46]
Jordan (As-Salt Region)	111	[47]
Russia	48	[48]
India (Aizawl district)	48.4	[49]
Germany	49	[50]
Sweden	90	[51]
Spain	95	[52]
Greece	55	[53]
France	89	[53]
Iceland	13	[54]
Ireland	77	[55]
Romania	126	[56]
Nigeria (Southwest regions)	39	[57]
Ghana (South Dayi District)	34.9	[58]
Ecuador	94.3	[59]
Venezuela	52.5	[59]
Peru	32.29	[59]

## Calculation of Annual effective dose, ELCR and LCC associated with radon exposure

The annual effective dose by the indoor air radon was estimated by the following equation:

Annual effective dose (DT) = CR × D ×H × F × T (mSv/yr) [9,18,19].

Where: CR = Radon concentration (Bq/m^3^); D = Dose conversion factor (9 × 10^−6^ mSv/hr per Bq/m^3^); H = Indoor occupancy factor (0.8); F = Indoor radon equilibrium factor (0.4); and T = Number of hours in a year (24 h × 365 days =8760 h/yr).

The Annual effective dose (to lungs) was obtained by equation 2:

Annual effective dose (ET) to lungs = DT × WR × WT

DT = annual absorbed dose (mSv/yr); WR = radiation weighting factor (20 for alpha particles recommended by the ICRP); and WT = tissue weighting factor (0.12 for lung) [20]

The Excess life time cancer risk (ELCR) was calculated using the Equation 3:

ELCR = DT × DL × RF [21]

Where DT is the annual effective dose, DL is the average duration of life estimated to a 70 years and RF is the fatal cancer risk per Sievert (5.5 × 10^−2^ Sv^−1^) recommended by ICRP 103.

Finally, the lung cancer cases per year per million person (LCC) is estimated by using the risk factor lung cancer induction 18 × 10^−6^ mSv^-1^ and can be obtained using the Equation 4:

LCC = DT×18 × 10^−6^ [17,22].

According to the Table 1 the values of annual effective doses for radon inhalation by the inhabitants were found to vary in the range 0.28 (Kermanshah) to 11.07 (Ramsar) mSv y^–1^ with a mean of 2.6 ± 2.4 mSvy^–1^. It has been observed that majority of the cities monitored for indoor radon concentration were shown annual effective dose within the recommended action level (3–10 mSvy^−1^) [23].

**Fig. 2:**
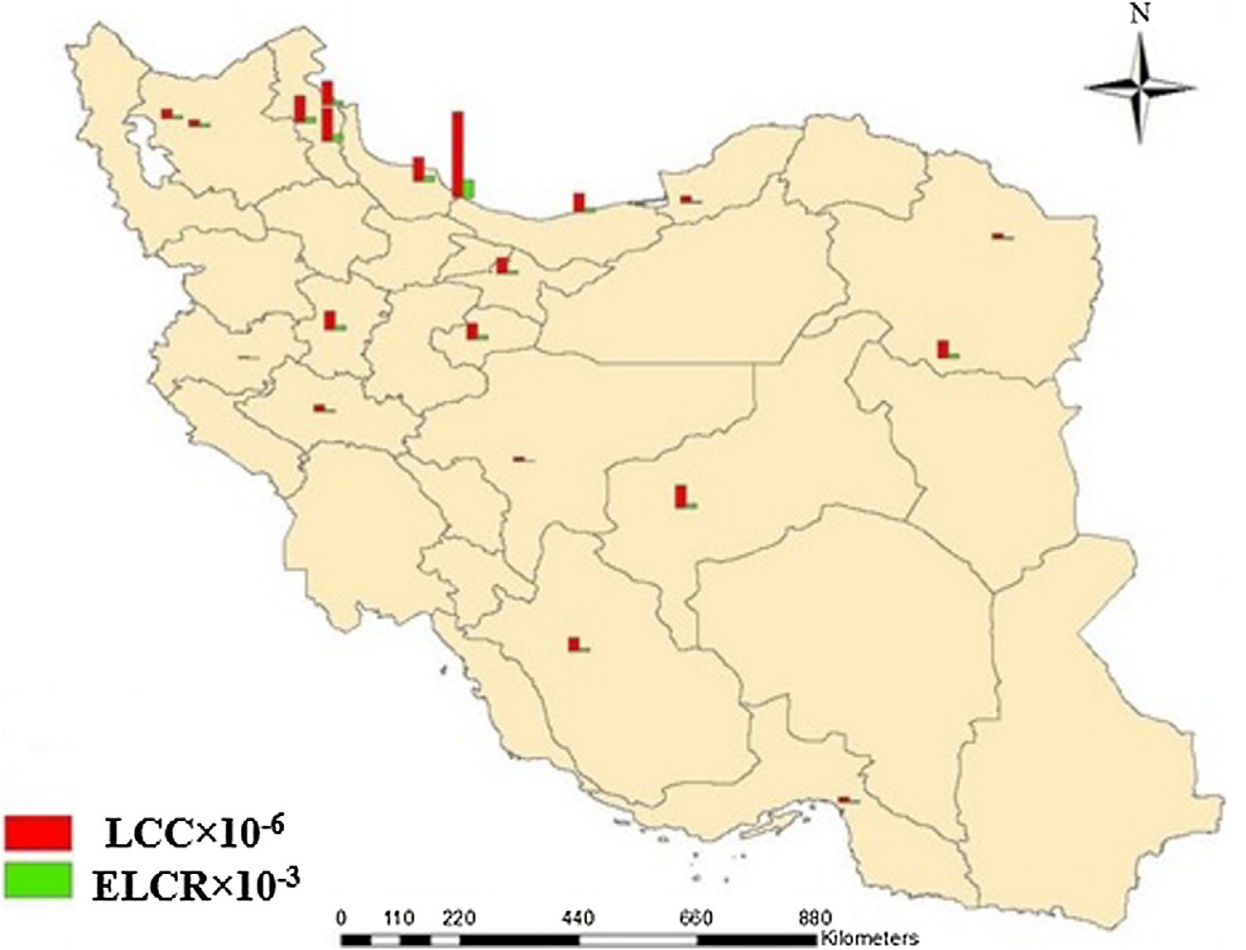
Indoor radon risk map of Iran.

The ELCR and risk of lung cancer estimated from 3441dwellings surveyed are presented in Fig. 2.

It is found that LCC ranged between 5.04 and 199.2 per million persons per year with mean value of 46.8 per million persons per year which is lower than the limit range of 170–230 per million persons recommended by ICRP [24].

## Case survey: Shabestar County

### Study area and data collection

Shabestar County is located in 45° 05′ to 46° 09′ eastern longitudes and 37° 42′ to 38° 24′ northern latitudes is a county in East Azerbaijan province in Iran. It is limited to Tabriz city and Urmia Lake from northwest and northeast respectively. The climate of study area is mostly semiarid and the minimum and maximum of temperature in the area are −14 °C in winter and +31 °C in summer, respectively [25,26].

**Fig. 3:**
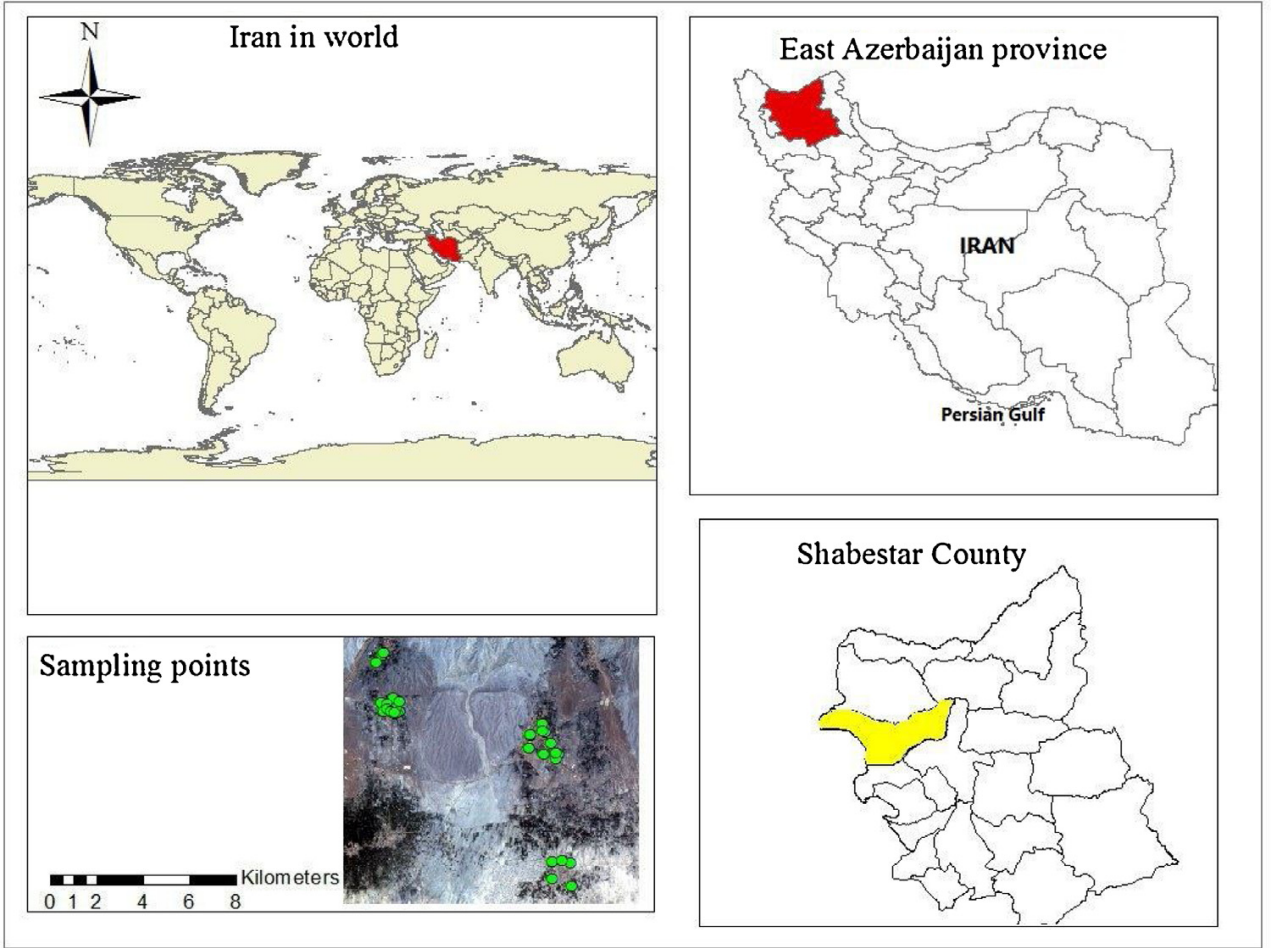
Location Map of the Study Area.

This cross-sectional study was carried out during winter of 2016 on 50 residential houses which were randomly selected with an emphasis on coverage of whole investigated area from Shabestar, Khamaneh, Vayqan and Daryan cities. The location of study area and sampling points has been shown in Fig. 3.

A passive sampling using solid-state nuclear detectors of SSNTDs type CR-39 was performed to measure the concentration of radon gas. CR-39 polycarbonate film placed inside a plastic holder. Detectors were numbered and for each building unit, two detectors were installed in the bedroom and living room and left for a period of three months while placed at a distance of 90 cm from the floor and away from sunlight and windows.

After three months of exposure, detectors were wrapped with aluminum foils and transferred to the Reference Radon Lab, Mazandaran University of Medical Sciences. In the laboratory, then detectors placed in a 6.25 N solution of NaOH at 85 °C for 3 h due to magnify the alpha tracks. The films were washed with distilled water after the time was spent to read. The automatic counting system with taking microscope images was used for counting alpha particles (tracks) recorded on CR-39 detectors, then using calibration and conversion factors track density was converted to the radon concentration in Bq/m^3^. All the radon determination process was carried out according to the U.S. EPA protocol [1,27].

In order to evaluation of affecting parameters on radon levels, the information about type of building (villa or apartment), floor numbers, the age of the building, the type of skeleton construction materials and cracking on the wall and roof were gathered and recorded.

Statistical analysis of the data was performed by SPSS version 20 and Excel v. 2016. The variables were normalized with the log-transformation and analyzed using parametric tests.

The average concentration of indoor radon in Shabestar, Khamaneh, Vayqan and Daryan buildings were (in order) 43.53 ± 26.93, 63.18 ± 83.4, 67.11 ± 50.16 and 63.25 ± 26.34 Bq/m^3^. Comparing the arithmetic mean indoor radon concentration of this work to other studies done across the country reveals that Shabestar county has a lower mean indoor radon (56.19 ± 45.96 Bq/m^3^), than means (117.4 ± 97.7 Bq/m^3^) obtained in Table 1. However, the mean value is higher than the global average (40 Bq/m^3^) [28].The maximum measurement was 520.12 Bq/m^3^ (approximately 3.5 times higher than the limit imposed by EPA) in bedroom (1^st^ floor) of a 5-year age building with granite stones in facade and some artificially fashioned building materials (Patina) in rooms.

The minimum and maximum values in bedrooms amounted to 3.92 and 520.12 Bq/m^3^ while the concentration of radon gas in living rooms was ranged from 4.94 to 155.02 Bq/m^3^ in the studied area. Despite the large variations in bedrooms than living rooms, there was no statistical significant difference between radon concentration in these environments (p > 0.05(. The results of Pearson correlation analysis between the indoor radon and some affecting variables in radon emission was presented in Table 3 and confirmed a good linear relationship between bedroom and living-room radon concentration.

**Table 3:** Pearson Correlation analysis between bedroom and living-room radon with other factors.

Variables	Living room	Bed room	Building type	Floor number	Building facade’s	Building age	Building structure	Floor covering	Wall covering	Window type	crack
Living room	1										
Bedroom	.448**	1									
Building type	.353*	.336*	1								
Floor number	−0.053	−0.294	−.509**	1							
Building facade’s	0.193	−0.121	0.161	0.033	1						
Building age	.455**	0.05	0.327	0.132	0.136	1					
Building Structure	0.238	0.169	0.204	0.082	0.053	.365*	1				
Floor covering	−0.01	0.052	−0.192	0.132	−0.075	−0.291	0.042	1			
Wall covering	0.07	0.059	.363*	−0.204	0.093	.360*	0.101	−0.279	1		
Window type	−0.026	−.322*	−0.254	0.177	−0.097	0.104	−0.071	0.163	−0.2	1	
Crack	.373*	.333*	0.122	0.21	0.155	0.29	0.106	−0.188	−0.007	−0.171	1

Although, some moderate associations were detected with the factors “building age, cracks on the walls/floor, window type”; nonetheless the pairwise regression analysis confirmed no strong correlation between them and radon concentration in bedroom and living rooms.

**Fig. 4:**
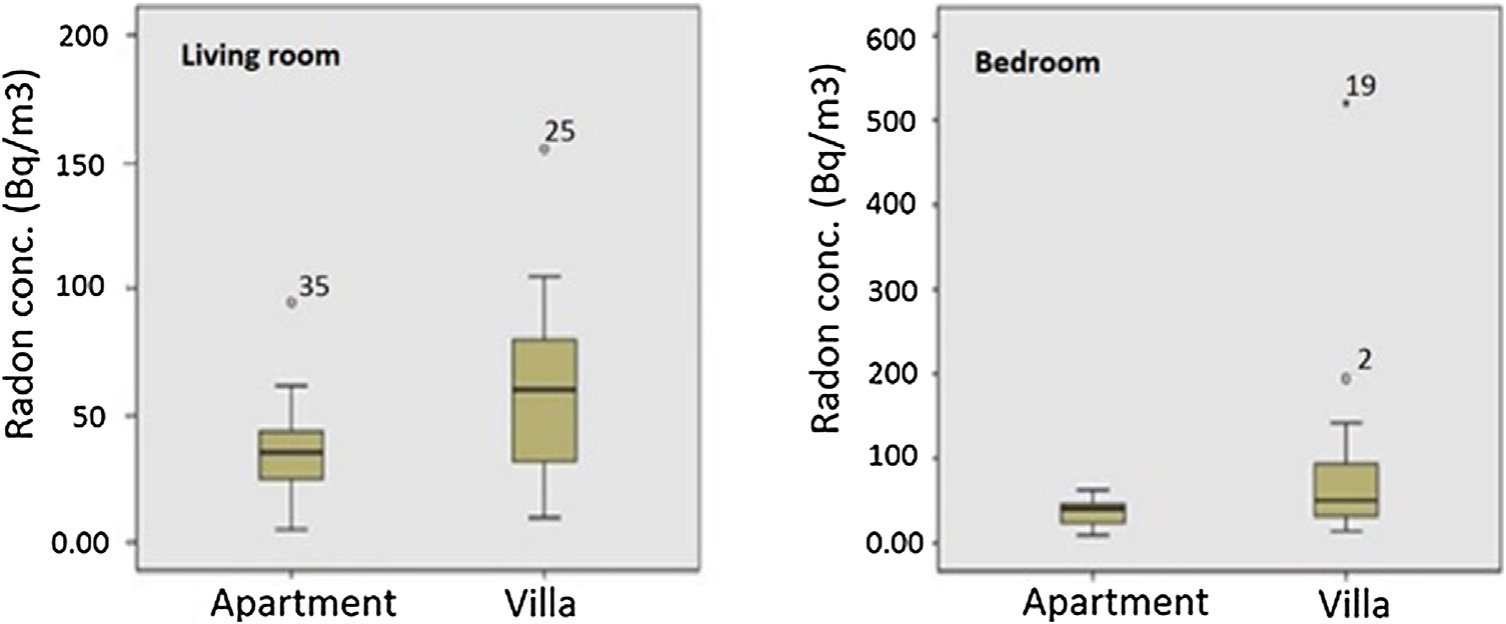
Radon concentration variations with the type of building.

Also, a significant difference was observed between radon levels in apartment (multi-story homes) and villa dwellings (*p* < 0.05). According to the Fig. 4, radon content was higher in villa relative to apartments The average radon content was found to be 65.51 Bq/m^3^ for the villa and 34.22 Bq/m^3^ for the apartments. Because radon is heavier than air, it tends to sink to the lowest possible levels of homes and also its concentrations are appreciably high in isolated houses than in blocks of apartments [29]. As reported in some other studies [14,[34], [35], [36]].

### Radiation dose estimation

The mean annual effective doses resulting from the radon gas in different floors of dwellings in Shabestar County are shown in Table 4. The results comparison with data obtained from other parts of the country (See Table 1) suggests that the average mean value for Shabestar County is less than the calculated mean effective dose rates for other parts of Iran (2.6 ± 2.4 mSv.y^−1^).

**Table 4:** Indoor radon concentration and their respective doses at different floor.

	Rn concentration (Bq/m^3^)		D_T_)mSv/y(
1 st floor	Bedroom	60.41	1.52
	Living room	52.14	1.32
	Average	60.89	1.54
2nd and upper floors	Bedroom	56.65	1.43
	Living room	51.58	1.3
	Average	41.15	1.04

The mean annual effective dose and effective dose (to lungs) received by the residents of the studied area in Shabestar county were estimated to be 1.4 and 3.5 mSv/y respectively (Table 5). The mean ELCR for indoor exposure in the area was found to be 0.54/100 people that is small as compared with action level of EPA, the estimated risk of 1.3% corresponding with radon exposure of 148 Bq/m^3^ for the entire population [32].

**Table 5:** The ^222^Rn Concentration, Absorbed dose and Effective Dose to Lungs for studied area.

Location	Average radon concentration (Bq/m^3^)	Annual effective dose(mSv/y)	Annual effective dose to lungs(mSv/y)
Total studied areas	56.19	1.4	3.36
Shabestar	44.46	1.12	2.69
Khamaneh	65.38	1.65	3.96
Vayqan	60.93	1.53	3.67
Daryan	65.89	1.66	3.99

The annual average effective doses received by residents in the villa homes (1.65mSv.y^−1^) are about two times the doses received in the apartments (0.83mSv.y^−1^).

## Additional information

Soil, uranium and phosphate mines, and coal combustion can be considered as the main sources of radon release to the environment. Outdoors radon levels are generally low. Indoors radon levels, in buildings and homes especially in basements and lower floors can become much higher because of radon entrance through cracks and openings in the foundation. Health effects of radon are well known [33]. Exposure to radon often occurs primarily from breathing radon in air, resulting in an increase in the incidence of lung cancer due to damage DNA of cells lining the airways. Exposure to radon is the second leading cause of lung cancer. The World Health Organization (WHO) has estimated that in a country, between 3–14% of all lung cancers can be attributed to radon. This percent depends on the national average radon level and smoking prevalence [34].

To the best of our knowledge, no attempts have been made to harmonize data on the distribution of indoor radon concentration and mapping at the country level so far. To assess and compare data measured in different cities, we produced a preliminary map but it is evident the reported data is sketchy and local surveys are still ongoing. The average radon activity concentration less than 100 Bq/m^3^ was found in 64% of studied areas in Iran and no more than 10% of them have radon concentration exceeding 200 Bq/m^3^. Also, the geometric mean of radon concentration was calculated to 72.05 Bq/m^3^.

Considering Shabestar County, the radon data recorded in dwellings (mean: 56.19 ± 45.96 Bq/m^3^) is less than prescribed levels by WHO, EPA, ICRP and as well values averaged at the level of country (117.4 ± 97.7 Bq/m^3^). Also, the value of the annual effective dose ranged from 1.12 to 1.66 mSv.y^−1^ with a mean value of 1.4 mSv.y^−1^, which is below even the lower limit of the recommended action level of 3–10 mSv.y^−1^.

Due to complexity of influential factors and variability of radon gas levels, conducting further studies especially on radon release sources, local geology and soil-gas radon concentration, radon concentration in drinking water and estimation of annual ingestion and inhalation doses is recommended.

## Declaration of Competing Interest

The authors declare that no conflict of interest exists in publishing this article.
